# Evaluation of an On-Farm Culture System (Accumast) for Fast Identification of Milk Pathogens Associated with Clinical Mastitis in Dairy Cows

**DOI:** 10.1371/journal.pone.0155314

**Published:** 2016-05-13

**Authors:** Erika Korzune Ganda, Rafael Sisconeto Bisinotto, Dean Harrison Decter, Rodrigo Carvalho Bicalho

**Affiliations:** Department of Population Medicine and Diagnostic Sciences, College of Veterinary Medicine, Cornell University, Ithaca, NY, United States of America; University of British Columbia, CANADA

## Abstract

The present study aimed evaluate an on-farm culture system for identification of milk pathogens associated with clinical mastitis in dairy cows using two different gold standard approaches: standard laboratory culture in study 1 and 16S rRNA sequencing in study 2. In study 1, milk from mastitic quarters (i.e. presence of flakes, clots, or serous milk; n = 538) was cultured on-farm using a single plate containing three selective chromogenic media (**Accumast—**FERA Animal Health LCC, Ithaca, NY) and in a reference laboratory using standard culture methods, which was considered the gold standard. In study 2, mastitic milk was cultured on-farm and analyzed through 16S rRNA sequencing (n = 214). In both studies, plates were cultured aerobically at 37°C for 24 h and read by a single technician masked to gold standard results. Accuracy, sensitivity, specificity, positive (**PPV**) and negative predictive value (**NPV**) were calculated based on standard laboratory culture in study 1, and PPV was calculated based on sequencing results in study 2. Overall accuracy of Accumast was 84.9%. Likewise, accuracy for identification of Gram-negative bacteria, *Staphylococcus* sp., and *Streptococcus* sp. was 96.4%, 93.8%, and 91.5%, respectively. Sensitivity, specificity, PPV, and NPV were 75.0%, 97.9%, 79.6%, and 97.3% for identification of *E*. *coli*, 100.0%, 99.8%, 87.5%, and 100.0% for *S*. *aureus*, 70.0%, 95.0%, 45.7%, and 98.1% for other *Staphylococcus* sp., and 90.0%, 92.9%, 91.8%, and 91.2% for *Streptococcus* sp. In study 2, Accumast PPV was 96.7% for *E*. *coli*, 100.0% for *Enterococcus* sp., 100.0% for Other Gram-negatives, 88.2% for *Staphylococcus* sp., and 95.0% for *Streptococcus* sp., respectively. In conclusion, Accumast is a unique approach for on-farm identification pathogens associated with mastitis, presenting overall sensitivity and specificity of 82.3% and 89.9% respectively.

## Introduction

Clinical mastitis remains an important animal health issue and leads to major economic losses to the dairy industry worldwide. From 20% to 30% of dairy cows are diagnosed with clinical mastitis at least once during lactation [1, 2]. Estimated costs per case of clinical mastitis range between $179 and $488 depending upon milk prices, level of production in affected cows, culling policies, and stage of lactation when the disease occurred [3, 4]. In fact, treatment and prevention of mastitis are considered the most common causes of antibiotic use in dairy herds [5, 6]. Although fungi and algae have been observed in the milk of cows diagnosed with clinical mastitis, inflammation of the mammary gland is caused predominantly by bacterial infections. *Staphylococcus* sp., *Streptococcus* sp., and coliforms account for approximately 90% of isolates in the milk of mastitic cows [1, 7]. Of particular importance for mastitis control programs, the success of antimicrobial therapy is dependent upon the causal pathogen associated with clinical mastitis. Intramammary antibiotic therapy improves the rate of cure in cows infected with coagulase-negative staphylococci, *Staphylococcus* sp., and environmental streptococci [8]. On the other hand, the use of an intramammary antibiotic is not recommended for cows with mastitis associated with *E*. *coli* [9]. Rapid on-farm identification of milk pathogens is critical for targeted antimicrobial therapy, which helps avoid the indiscriminate use of antibiotics in livestock and reduces the economic burden of clinical mastitis.

Several on-farm culture systems have been developed to characterize milk pathogens and substantiate the decision to treat cows with clinical mastitis. Initial on-farm culture systems were based on blood and MacConkey agar plates, which allowed for categorization of microorganisms into Gram-positive, Gram-negative, or no growth within 24 to 32 h and at relative low cost compared to the use of referral diagnostic laboratories. Previously published studies indicate that the use of selective treatment based on on-farm culture systems results has the potential to reduce antibiotic usage by 50% with no changes in the risk of disease recurrence, bacteriological cure, somatic cell count, milk production, and survival throughout lactation [10, 11]. More sophisticated on-farm culture systems allow for further genus classification of Gram-positive bacteria into *Staphylococcus* sp. and *Streptococcus* sp. and evaluation of *Staphylococcus aureus* presence in milk [12]. Nevertheless, using these other techniques the assessment of colony appearance is required for detailed identification of milk pathogens and may reduce the predictive value of on-farm culture systems when conducted by farm personnel. In fact, the sensitivity of selective culture media to detect *S*. *aureus* in milk samples ranged from 43.2% to 59.1% when plates were read by individuals with only limited microbiology training [13].

Chromogenic media have been used for identification of microorganisms in both human and animal specimens [14, 15]. Chromogens incorporated in the culture media are cleaved by specific bacterial enzymes generating chromophores, which can be readily recognized with the naked-eye based on color change. The use of chromogenic culture media for identification of milk pathogens associated with mastitis has not been previously evaluated. However, this technology has the potential to increase the number of bacteria distinguishable using on-farm culture systems without requiring intensive microbiology training by farm personnel. The main hypothesis of this study was that the use of chromogenic culture media allows for identification of milk pathogens associated with clinical mastitis in lactating dairy cows with satisfactory sensitivity and specificity. Therefore, specific objectives were to evaluate the use a of selective chromogenic on-farm culture system designed for identification of specific mastitis pathogens: staphylococci, streptococci, and Gram-negative bacteria, constituted by a single plate containing three selective chromogenic media (**Accumast**—FERA Animal Health LCC, Ithaca, NY). Predictive values of this on-farm culture system were evaluated based on the results from an official diagnostic laboratory (study 1) and on through molecular identification of cultured pathogens using 16S rRNA sequencing (study 2) as gold standards.

## Materials and Methods

### *In-vitro* assessment of colony characteristics of pure bacterial cultures plated onto chromogenic media

**Fig 1:**
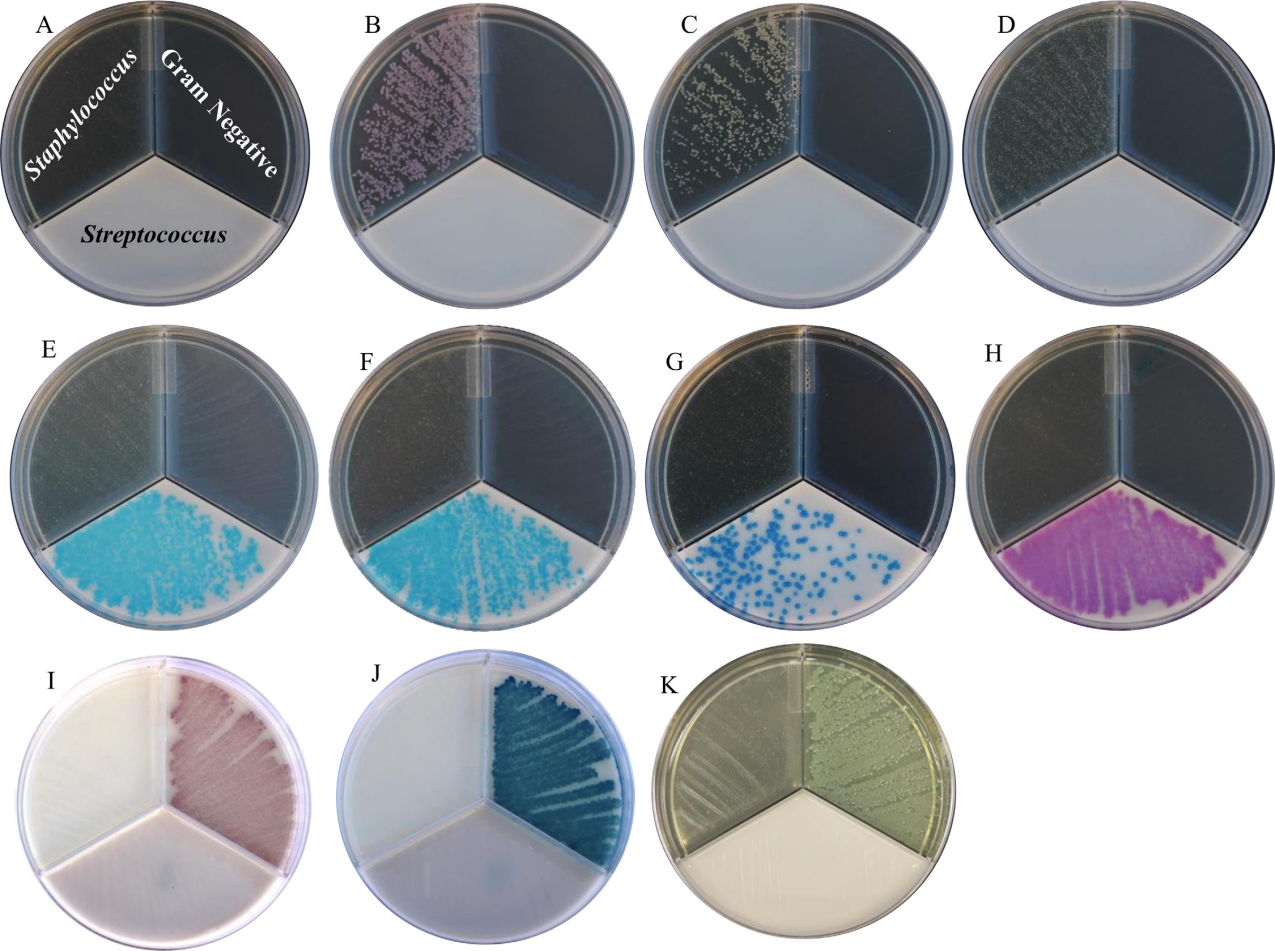
Visual assessment of Gram-positive and Gram-negative bacterial growth on Accumast plates performed in laboratory. Pictures were taken onto dark background. Plate without bacteria (panel A), *Staphylococcus aureus* (panel B), *Staphylococcus epidermidis* (panel C), *Staphylococcus chromogenes* (panel D), *Streptococcus agalactiae* (panel E), *Streptococcus dysgalactiae* (panel F), *Streptococcus uberis* (panel G), *Enterococcus faecalis* (panel H) *Escherichia coli* (panel I)*, *Klebsiella oxytoca* (panel J)*, and *Pseudomonas aeruginosa* (panel K). *Pictures were taken onto light background.

The assessment of growth of pure ATCC strains of pathogens previously described to be associated with bovine mastitis was performed in laboratory using Accumast for the purpose of evaluation of the growth characteristics. The following Gram-positive and Gram-negative ATCC strains (Species, catalog number) were used in the evaluation: (*Staphylococcus aureus*, 25923; *Staphylococcus epidermidis*, 12228; *Staphylococcus chromogenes*, 43764; *Streptococcus agalactiae*, 27956; *Streptococcus dysgalactiae*, 43078; *Streptococcus uberis*, 700407; *Enterococcus faecalis*, 29212; *Escherichia coli*, 25922; *Klebsiella oxytoca*, 49131; *Pseudomonas aeruginosa*, 15442). Bacteria stocks were activated in unselective tryptic soy agar plates supplemented with 5% sheep blood and 0.1% esculin (BioMerieux, Durhan, NC). Plates were incubated aerobically at 37°C for 24 h to ensure the presence of live bacteria and absence of contamination. For each strain, a single colony was transferred to 5 mL of brain heart infusion broth (Bacto Brain Heart Infusion; Becton, Dickinson and Company, Franklin Lakes, NJ), homogenized, and incubated overnight aerobically at 37°C. Bacterial cultures were diluted to 1:1,000 in sterile PBS solution (Boston BioProducts, Ashland, MA). A sterile cotton swab was used to plate the diluted bacterial culture into each section of Accumast, ensuring that the swab had been saturated in the bacterial sample between plating different sections of the plate. Plates were incubated aerobically at 37°C (Fig 1).

The threshold selected for considering a sample positive for bacterial growth using the Accumast culturing system was the presence of five or more colonies in a single section of the plate. Presence of bacterial growth in each of two different sections of the plate was considered a mixed infection and counted as positive for both types of bacteria. Presence of bacterial growth in each of three sections was considered contamination.

### Ethics statement

The present study was carried out in agreement with the recommendations of The Animal Welfare Act of 1966 (P.L. 89–544) and its amendments of 1970 (P.L. 91–579), 1976 (P.L. 94–279), and 1985 (P.L. 99–1998) that regulates the transportation, purchase and treatment of animals used in research. The research protocol was reviewed and approved by the Institutional Animal Care and use Committee of the Cornell University (Protocol number: 2013–0056). Sampling animals that present abnormal milk during forestripping on milking preparation is also routine procedure at the study site.

### Conflict of interest statement

The product evaluated in the present study was originally developed in Dr. Bicalho’s research laboratory, in Cornell University, Ithaca, NY. Cornell University requires inventors to assign to the university or its designee all rights and titles of their inventions and related property rights that result from activity conducted in the course of an appointment with the university and/or using university resources, including those provided through an externally funded grant, contract, or other type of award or gift to the university. A U. S. Provisional Patent application (No. 62/212,482) was submitted by Cornell University on August 31, 2015 and listed Dr. Bicalho as the inventor. Dr. Bicalho founded the company FERA Animal Health, LLC and licensed the patent rights from Cornell University on February 25, 2016.

### Farm and management

On-farm evaluation of the use of Accumast for identification of pathogens associated with clinical mastitis was performed in a single commercial dairy herd located in Venice Center, NY. During the study, approximately 2,800 cows were milked thrice daily in a double-52 milking parlor and the yearly rolling herd average for milk yield was 13,800 kg, with an average bulk tank somatic cell count of 360.000 cells/mL. Primiparous and multiparous cows were housed separately in free-stall barns equipped with sprinklers, fans, and concrete stalls bedded with manure solids. Cows were fed a total mixed ration to meet or exceed the nutrient requirements of a 650 kg lactating Holstein cow producing 45 kg/d of milk with 3.5% fat and 3.2% true protein when dry matter intake is 25 kg/d [16].

### Sample collection

Clinical mastitis was defined as the presence of abnormal milk (i.e. presence of flakes, clots, or serous milk) during forestripping performed at the milking parlor. Milk from affected quarters was sampled aseptically by trained farm personnel following recommendations of the National Mastitis Council. Briefly, teats were cleaned and disinfected using 70% ethanol (vol/vol). The initial three streams were discarded and approximately 5 mL of milk was collected into a sterile plastic tube without preservative (Corning Life Sciences, Tewksbury, MA). Milk samples were kept refrigerated at 4°C in a designated office adjacent to the milking parlor and plated at the farm no longer than 12 h after sample collection.

### On-farm culture system

The on-farm culture system evaluated here was created to allow for selective growth and identification of specific mastitis pathogens (i.e. staphylococci, streptococci, and Gram-negative bacteria), using a single plate containing three selective chromogenic media (Accumast, FERA Animal Health LCC, Ithaca, NY).

**Fig 2:**
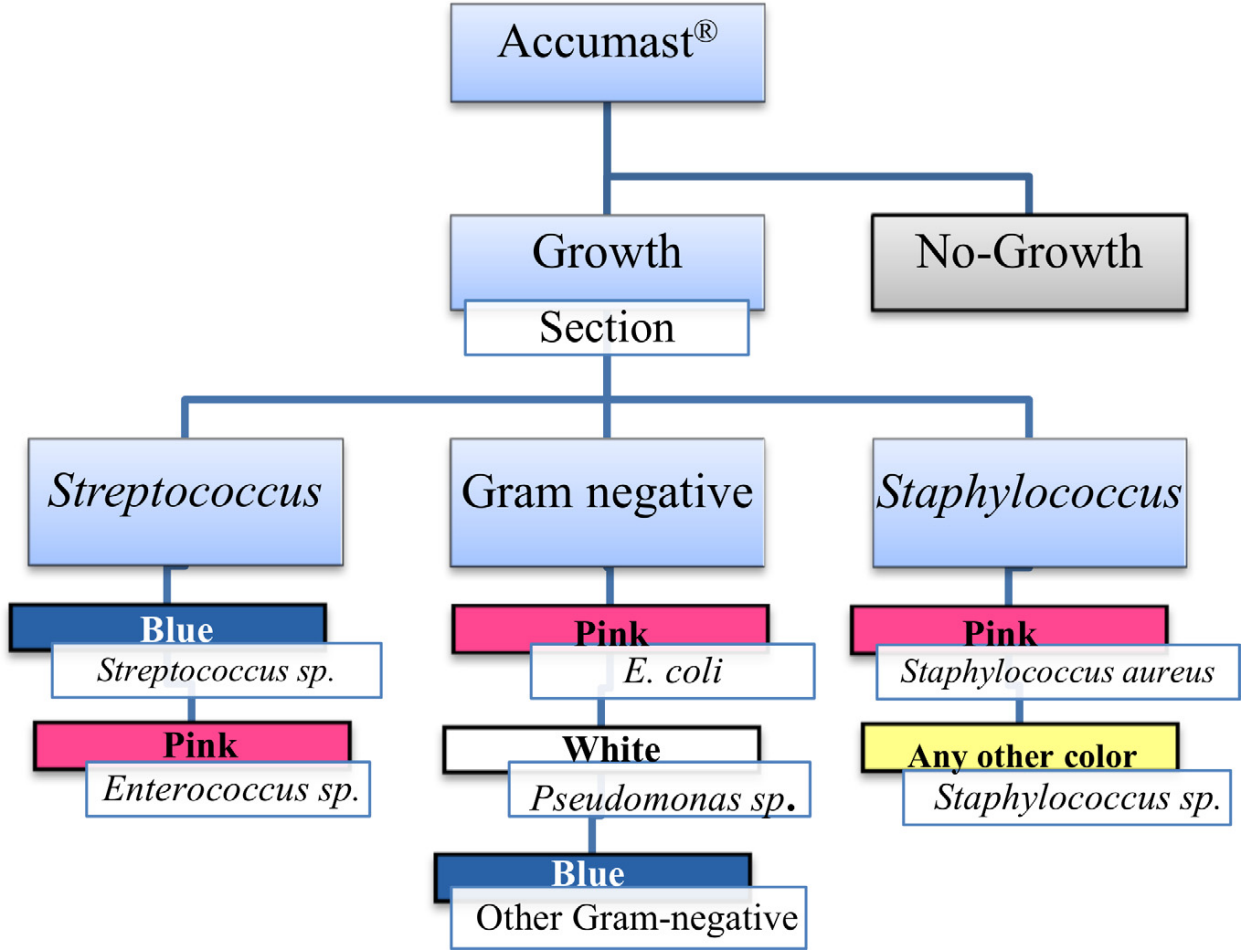
Flow-chart for on-farm diagnosis of mastitis pathogens based on Accumast.

Milk samples were plated onto Accumast using a sterile cotton swab. Before application into each of the three sections of Accumast the swab was immersed in the milk sample. Plates were incubated at 37°C for 24 h and read on-farm by one of the investigators according to the flowchart provided for on-farm diagnosis of mastitis pathogens identifiable by Accumast (Fig 2). Presence of bacterial growth, number of colonies, and color of colonies were recorded. Identification of milk pathogens was performed following instructions of the flowchart developed based on characteristics of growth of American Type Culture Collection (**ATCC**) strains described above.

### Study 1: Identification of milk pathogens by standard laboratory culture

A total of 538 milk samples obtained from cows affected with clinical mastitis were collected as described above, from April to July, 2014. Immediately after collection and plating onto Accumast, milk samples were refrigerated at 4°C and transported to the Quality Milk Production Services (**QMPS**) laboratory (Cornell University, Ithaca, NY) on ice. Milk samples were cultured following standard procedures for mastitis associated pathogens identification [17] and standard laboratory culture results were used as gold standard for evaluation of on-farm culture system. In summary, milk samples were plated onto trypticase soy agar plates supplemented with 5% of sheep blood and 0.1% esculin using a sterile cotton swab. Plates were incubated aerobically at 35°C to 38°C for at least 24 h but no longer than 48 h. Culture characteristics evaluated included size, color, hemolytic pattern, and odor. Ancillary tests for further bacterial classification included Gram stain and wet mount microscopic evaluations. Biochemical tests comprised evaluation of the presence of catalase using 3% hydrogen peroxide, coagulase using EDTA rabbit plasma tubes, indole using SpotTest (Hardy Diagnostics), KOH string test using 3% potassium hydroxide, oxidase, lactose, sorbitol fermentation, and CAMP tests. Additionally, surface carbohydrates group typing (BactiStaph and PathoDx, Thermo Scientific) and selective differential agars such as MacConkey, Edwards, and bile esculin were used when needed. Samples were considered mixed infections when two clearly distinct bacterial types in a well distributed growth pattern were detected, and both pathogens were reported. Identification of more than two distinct colony types and no contagious pathogens such as *S*. *aureus* or *S*. *agalactiae* present was considered contamination of the sample. Samples were considered negative when no aerobic bacterial growth was observed in the first 48 h of incubation following guidelines for accredited diagnostic laboratories.

### Study 2: Identification of milk pathogens by 16S rRNA sequencing

To further evaluate the accuracy of Accumast for on-farm identification of milk pathogens, a second study utilized bacterial 16S rRNA sequencing from pathogens isolated from mastitic milk samples cultured on-farm between October to December of 2014 using Accumast plates as described above (n = 214) utilizing the Illumina platform.

On-farm identification of milk pathogens was performed as previously described. Plates were read at the farm and those with bacterial growth were transported at room temperature to the laboratory for bacterial isolation, DNA extraction, and sequencing. For each sample, one colony was selected from Accumast, collected using a sterile inoculating loop, and plated onto unselective tryptic soy agar plates supplemented with 5% sheep blood and 0.1% esculin (BioMerieux, Durhan, NC). Plates were incubated aerobically at 37°C for 24 h. This procedure was repeated twice using blood agar plates (Northeast Laboratory Services, Winslow, ME) to ensure that the colony isolated from Accumast was free of contamination.

Genomic DNA was isolated using InstaGene matrix (Bio-Rad laboratories, Hercules, CA) according to manufacturer’s recommendations. Concentration and purity of extracted DNA were evaluated based on optical density using a NanoDrop ND-1000 spectrophotometer (NanoDrop Technologies, Rockland, DE). Amplification of the V4 hypervariable region of the bacterial 16S rRNA gene was performed from genomic DNA by PCR utilizing the primers 515F (AATGATACGGCGACCACCGAGATCTACACTATGGTAATTGTGTGCCAGCMGCCGCGGTAA) and barcoded 806R (CAAGCAGAAGACGGCATACGAGAT*XXXXXXXXXXXX* AGTCAGTCAGCCGGACTACHVGGGTWTCTAAT) which have been optimized for the Illumina MiSeq platform [18]. To allow for multiplex sample sequencing, each sample was tagged with a unique 12-bp error-correcting Golay barcode for the 16S rRNA PCR selected using the earth microbiome project (http://www.earthmicrobiome.org/) as formerly described [18, 19]. Barcoded amplicons were generated in triplicate using 3 μL DNA template, 1X EconoTaq Plus Green Master Mix (Lucigen, Middleton, WI), and 5 μM of each primer. Thermocycler conditions were as follows: an initial denaturing step at 94°C for 3 min, 35 cycles of 94°C for 45 s, 50°C for 1 min, and 72°C for 90 s, and a final elongation step of 72°C for 10 min. Blank controls were incorporated in each reaction batch to ensure the absence of bacterial DNA contamination. Replicate amplicons were pooled and and visualized by electrophoresis through 1.2% (wt/vol) agarose gels stained with 0.5 mg/mL ethidium bromide. Purification of amplicons was performed using Gel PCR DNA Fragment Extraction kit (IBI Scientific, Peosta, IA). The NanoDrop ND-1000 spectrophotometer was used for quantification.

Aliquots of 16S rRNA amplicons were standardized to the same concentration (i.e. 16 ng/μL) and sequentially diluted to 20 pM following DNA denaturation. Because bacterial DNA was harvested from a single purified colony, amplicons were combined with 70% of PhiX (Illumina Inc., San Diego, CA). Amplicons were pooled into a single run and final equimolar library was sequenced using the MiSeq reagent kit V2 Nano for 300 cycles on the MiSeq platform (Illumina Inc., San Diego, CA). The 16S rRNA gene sequences were processed using the MiSeq Reporter analysis software version 2.5. Indexed reads were demultiplexed for generation of individual FASTQ files and reads were aligned to the Illumina-curated version of Greengenes database for genus-level classification of milk pathogens.

### Statistical analyses

The predictive values of Accumast for the identification of pathogens associated with mastitis were evaluated based on comparing Accumast results with those from standard laboratory culture and results from 16S rRNA sequencing, which were considered the gold standards for comparison in study 1 and 2, respectively. Samples considered contaminated either by the reference laboratory (n = 3) or Accumast (n = 6) were not included in the analysis. Results are presented as parameter estimates and 95% confidence intervals. Confidence intervals were calculated based on the standard error obtained from a binomial distribution following the formulas: SE = $\frac{\sqrt{p(1-p)}}{n}$ and CI = estimate ± 1.96 × SE.

#### Study 1: Standard laboratory culture as gold standard

Sensitivity, specificity, positive predictive value (**PPV**), and negative predictive value (**NPV**) were calculated based on true positives, true negatives, false positives and false negatives as stated by [20] comparing results from on-farm culture of milk samples collected between April and July 2014 and reference laboratory results using the same milk samples. In addition, accuracy was calculated by dividing the number of true positives and true negatives by the total number of tests. The simple Cohen's kappa coefficient (*κ)* was calculated using the FREQ procedure of SAS version 9.3 (SAS/STAT, SAS Institute Inc., Cary, NC). This parameter assumes that the two response variables (on-farm culture system and gold standard) are independent ratings, and the coefficient equals 1 when there is complete agreement between the two tests. The null hypothesis for this test is that if agreement happens due to chance the Kappa coefficient is equal to zero. Under this null hypothesis, *P*-values associated with this test equal or smaller than 0.05 were considered significant.

#### Study 2: 16S rRNA sequencing as gold standard

Because only positive results from Accumast plates were analyzed by 16S rRNA sequencing, the PPV and the simple Cohen's kappa coefficient between sequencing and Accumast were assessed as previously described. Interpretation of Cohen's kappa coefficient was applied following description in [20]. A Kappa coefficient between 0.81 and 1.0 corresponded to almost perfect agreement, 0.61 to 0.80 represented substantial agreement, an estimate between 0.41 and 0.60 was considered moderate agreement and a value of 0.21 to 0.40 denoted fair agreements.

## Results

### Study 1: Prevalence of milk pathogens associated with clinical mastitis

The prevalence reported herein was calculated based on results from standard laboratory culture which was considered the gold standard and is known as the true prevalence. The most prevalent pathogens in the milk of cows diagnosed with clinical mastitis according to results from QMPS standard laboratory culture were *S*. *uberis*, *Streptococcus* sp., and *E*. *coli* (Table 1). Only a small proportion of quarters were diagnosed with mixed infections and 31.2% of milk samples did not result in growth of significant organisms. Three samples were characterized as contaminated by the reference laboratory and 6 samples were characterized as contaminated based on Accumast and were excluded from subsequent analyses. Detailed information is provided in Tables A and B in S1 File.

**Table 1:** Prevalence of pathogens associated with clinical mastitis.

Bacteria identified by laboratory culture^1^	Number	Prevalence, %
*Streptococcus uberis*	134	24.9
*Streptococcus* sp.	56	10.4
*Escherichia coli*	49	9.1
*Streptococcus dysgalactiae*	40	7.8
*Staphylococcus* sp.	28	5.2
*Klebsiella* sp.	16	3.0
Mixed infection	14	2.2
*Trueperella pyogenes*	10	1.9
*Staphylococcus aureus*	7	1.3
*Enterococcus* sp.	7	1.3
Gram-negative bacilli	5	0.9
*Pseudomonas* sp.	1	0.2
No growth	168	31.2
Contamination	3	0.6
Total	538	100

^1^ Results from standard laboratory culture performed by the Quality Milk Production Services laboratory at Cornell University (Ithaca, NY).

### Study 1: Test characteristics of Accumast plates for identification of milk pathogens compared to standard laboratory culture

The overall sensitivity, specificity, PPV, NPV, accuracy, and *κ* coefficient of Accumast for identification of mastitis associated pathogens are presented on (Table 2). Among the Gram-negative bacteria observed in milk samples, the sensitivity and PPV were smaller for the detection of other Gram-negatives compared with *E*. *coli* and *Pseudomonas* sp. (Table 3). The overall sensitivity, specificity, and accuracy for detection of *Staphylococcus* sp. were 78.4%, 94.9%, and 93.8%, respectively (Table 4). Nevertheless, the accuracy for the identification of *S*. *aureus* was greater than for other *Staphylococcus* sp. Additionally, overall sensitivity, specificity and accuracy for identification of bacteria belonging to the streptococci group was high and the Cohen’s kappa coefficient among Accumast and standard laboratory culture for this bacterial group was denoted substantial.

**Table 2:** Overall test characteristics of selective chromogenic culture plates to identify bacteria associated with clinical mastitis determined by standard laboratory culture.

Parameter	Accumast	95% Confidence Interval
Number of Tests	529	
True Prevalence, % (n/n)	66.2 (350/529)	(66.0–66.3)
Sensitivity, %	82.3	(82.1–82.5)
Specificity, %	89.9	(89.6–90.3)
PPV^1^, %	94.1	(94.0–94.3)
NPV^2^, %	72.2	(71.8–72.6)
Accuracy, %	84.9	(84.7–85.0)
*κ* ^3^, %	0.68	(0.61–0.74)
*κ P*-value	<0.0001	

Each milk sample was cultured for identification of bacteria associated with clinical mastitis and culture results from the reference laboratory were considered the gold standard. Each plate was capable of identifying Gram-negative bacteria (*E*. *coli*, *Pseudomonas* sp., and other Gram-negatives), *Staphylococcus* sp. (*S*. *aureus* and *Staphylococcus* sp.), and *Streptococcus* sp. Only sections with more than five colonies were considered positive. Samples considered contaminated in either standard culture (n = 3) or on-farm culture (n = 6) were not included in the analysis (n = 9). Plates with no bacterial growth (n = 168) were considered in all calculations.

^1^ Positive predictive value.

^2^ Negative predictive value.

^3^ Cohen’s kappa coefficient. *κ* ≤ 0 denotes poor agreement; 0.01 to 0.20 denotes slight agreement; 0.21 to 0.40 denotes fair agreement; 0.41 to 0.60 denotes moderate agreement; 0.61 to 0.80 denotes substantial agreement and 0.81 to 1.00 denotes almost perfect agreement.

**Table 3:** Test characteristics of Accumast plates to identify Gram-negative bacteria associated with clinical mastitis determined by standard laboratory culture.

	Plate results			
Parameter	Overall Gram-negative	*E*. *coli*	*Pseudomonas* sp.	Other Gram-negatives
True Prevalence, % (CI^1^) n/n	14.4 (14.2–14.5) 76/529	9.8 (9.7–9.9) 52/529	0.2 (0.2–0.2) 1/529	4.3 (4.3–4.4) 23/529
Sensitivity, % (CI)	81.6 (80.6–82.6)	75.0 (73.4–76.6)	100.0 (100.0–100.0)	52.2 (47.9–56.4)
Specificity, % (CI)	98.9 (98.9–98.9)	97.9 (97.8–98.0)	99.8 (99.8–99.8)	99.2 (99.2–99.2)
PPV^2^, % (CI)	92.5 (91.8–93.3)	79.6 (78.0–81.2)	50.0 (1.0–99.0)	75.0 (69.7–80.3)
NPV^3^, % (CI)	97.0 (96.9–97.0)	97.3 (97.2–97.4)	100.0 (100.0–100.0)	97.9 (97.8–97.9)
Accuracy, % (CI)	96.4 (96.3–96.5)	95.7 (95.6–95.7)	99.8 (99.8–99.8)	97.2 (97.1–97.2)
*κ*^4^, % (CI)	0.84 (0.77–0.91)	0.74 (0.65–0.84)	0.66 (0.04–1.0)	0.60 (0.41–0.78)
*κ P*-value	<0.0001	<0.0001	<0.0001	<0.0001

Each milk sample was cultured for identification of bacteria associated with clinical mastitis and culture results from the reference laboratory were considered the gold standard. Only sections with more than five colonies were considered positive. Samples considered contaminated in either standard culture (n = 3) or on-farm culture (n = 6) were not included in the analysis (n = 9). Plates with no bacterial growth (n = 168) were considered in all columns. Pure and mixed cultures with the species of interest were combined. Growth on Gram-negative section regardless of color of colonies was considered positive for overall calculations. Only correct identification of bacterial group based on color was considered positive for within group calculations.

^1^ 95% confidence interval.

^2^ Positive predictive value.

^3^ Negative predictive value.

^4^ Cohen’s kappa coefficient. *κ* ≤ 0 denotes poor agreement; 0.01 to 0.20 denotes slight agreement; 0.21 to 0.40 denotes fair agreement; 0.41 to 0.60 denotes moderate agreement; 0.61 to 0.80 denotes substantial agreement and 0.81 to 1.00 denotes almost perfect agreement.

**Table 4:** Test characteristics of Accumast to identify Gram-positive bacteria associated with clinical mastitis determined by standard laboratory culture.

	Plate results			
Parameter	Overall Gram-positive *Streptococcus*	Overall Gram-positive *Staphylococcus*	*S*. *aureus*	*Staphylococcus* sp.
True Prevalence, % (CI^1^) n/n	47.1 (46.9–47.3) 249/529	7.0 (6.9–7.1) 37/529	1.3 (1.3–1.4) 7/529	5.7 (5.6–5.8) 30/529
Sensitivity, % (CI)	90.0 (89.7–92.7)	78.4 (76.2–80.6)	100.0 (100.0–100.0)	70.0 (67.0–73.0)
Specificity, % (CI)	92.9 (92.7–93.0)	94.9 (94.8–95.0)	99.8 (99.8–99.8)	95.0 (94.9–95.1)
PPV^2^, % (CI)	91.8 (91.6–92.0)	53.7 (51.9–55.5)	87.5 (79.4–95.6)	45.7 (43.5–47.8)
NPV^3^, % (CI)	91.2 (91.0–91.4)	98.3 (98.3–98.4)	100.0 (100.0–100.0)	98.1 (98.1–98.2)
Accuracy, % (CI)	91.5 (91.4–91.6)	93.8 (93.7–93.9)	99.8 (99.8–99.8)	93.6 (93.5–93.7)
*κ*^4^, % (CI)	0.82 (0.78–0.87)	0.60 (0.48–0.72)	0.93 (0.80–1.00)	0.52 (0.37–0.66)
*κ P*-value	<0.0001	<0.0001	<0.0001	<0.0001

Each milk sample was cultured for identification of bacteria associated with clinical mastitis and culture results from the reference laboratory were considered the gold standard. Only sections with more than five colonies were considered positive. Samples considered contaminated in either standard culture (n = 3) or on-farm culture (n = 6) were not included in the analysis (n = 9). Plates with no bacterial growth (n = 168) were considered in all columns. Pure and mixed cultures with the species of interest were combined. Growth on Gram-positive section regardless of color of colonies was considered positive for overall calculations. Only correct identification of bacterial group based on color was considered positive for within group calculations.

^1^ 95% confidence interval.

^2^ Positive predictive value.

^3^ Negative predictive value.

^4^ Cohen’s kappa coefficient. *κ* ≤ 0 denotes poor agreement; 0.01 to 0.20 denotes slight agreement; 0.21 to 0.40 denotes fair agreement; 0.41 to 0.60 denotes moderate agreement; 0.61 to 0.80 denotes substantial agreement and 0.81 to 1.00 denotes almost perfect agreement.

### Study 2: Test characteristics of Accumast plates for identification of milk pathogens compared to 16S rRNA gene sequencing

Results from 16S rRNA gene sequencing confirmed the precision of Accumast plates for identification of milk pathogens associated with clinical mastitis in dairy cows (Table 5). Cohen’s kappa coefficient was above 85% for all bacterial groups evaluated. Likewise, PPV was above 88% across all groups.

**Table 5:** Test characteristics Accumast plates to identify bacteria associated with clinical mastitis determined by 16S rRNA sequencing.

	Parameter			
Bacterial group	Tests, (n/n)	PPV^1^, % (CI^2^)	*κ* ^3^, %	*κ P*-value
Overall	214	95.3 (95.1–95.5)	0.89 (0.83–0.95)	<0.0001
*Escherichia*	30/214	96.7 (95.5–97.8)	0.85 (0.76–0.95)	<0.0001
*Enterococcus*	3/214	100.0 (100.0–100.0)	1.000 (1.0–1.00)	<0.0001
Other Gram-negatives	23/214	100.0 (100.0–100.0)	0.95 (0.88–1.00)	<0.0001
*Staphylococcus*	17/214	88.2 (84.5–91.9)	0.93 (0.83–1.00)	<0.0001
*Streptococcus*	141/214	95.0 (94.7–95.3)	0.91 (0.86–0.97)	<0.0001

Isolates from cases of clinical mastitis cultured using Accumast plates from October 2014 to December 2014 were subjected to 16S rRNA gene sequencing for genus level determination of bacterial growth. Only positive results were available for comparison, therefore only PPV and the Cohen’s kappa coefficient between 16S rRNA sequencing and Accumast were calculated.

^1^ Positive predictive value.

^2^ 95% confidence interval.

^3^ Cohen’s kappa coefficient. *κ* ≤ 0 denotes poor agreement; 0.01 to 0.20 denotes slight agreement; 0.21 to 0.40 denotes fair agreement; 0.41 to 0.60 denotes moderate agreement; 0.61 to 0.80 denotes substantial agreement and 0.81 to 1.00 denotes almost perfect agreement.

## Discussion

Knowing the etiology of mammary infections is extremely valuable for the development of strategies to control mastitis [21]. Selective treatment of cows diagnosed with clinical mastitis present major advantages to dairy herds including smaller costs associated with antimicrobials, reduction in the number of animals managed in the hospital pen, less discarded milk, and a potential reduction in the rate of development of antibiotic resistance in livestock [10, 22]. Targeted therapy relies on rapid and accurate identification of milk pathogens [2, 21, 23, 24]. The on-farm culture system evaluated in this study was designed to enable farm personnel to identify major bacteria associated with clinical mastitis in dairy cows in a straightforward manner. The spectrum of microorganisms identifiable using Accumast encompasses both Gram-positive (i.e. streptococci, staphylococci, and *S*. *aureus*) and Gram-negative pathogens (i.e. *E*. *coli*, *Pseudomonas* sp. and other Gram-negative pathogens). These bacteria have been reported to represent 80% and 100% of all milk pathogens isolated from mastitic cows in the states of New York [25] and Wisconsin [26]. When compared to a referral laboratory, the overall accuracy of Accumast to identify milk pathogens was 84.9%; with individual accuracies of 96.4%, 93.8%, and 91.5% for Gram-negative bacteria, staphylococci, and streptococci, respectively. Additionally, when compared to 16S rRNA sequencing results, the overall positive predictive value of Accumast was 95.3% and the Cohen's kappa coefficient was 0.89, which according to [20], is considered almost perfect agreement. The present results support the use of this culture system for on-farm identification of pathogens associated with clinical mastitis, for decision making in targeted treatment protocols, and for pathogen prevalence surveillance. It is important to acknowledge that certain pathogens were present in very low prevalence in our study (e. g. *S*. *aureus)* and further research should be conducted to validate current findings.

Major advances in the control of contagious pathogens implicated in mastitis have been accomplished through improvement of milking hygiene and management practices [27–29]. However, mammary infections with *S*. *aureus* remain a concern in dairy herds and require constant surveillance, aggressive antibiotic therapy, and segregation or culling of infected cows [30, 31]. On-farm culture systems have been used successfully to characterize bacteria present in the milk of mastitic cows as Gram-positive, Gram-negative, or no growth after 24 h to 32 h of incubation. Nonetheless, inconsistent results were observed when classification at the genus and/or species level was attempted by readers lacking extensive microbiology training. For instance, the sensitivity and specificity of University of Minnesota Triplate for identification of *S*. *aureus* by four readers with limited microbiology training ranged from 43.2% to 59.1% and 93.8% to 95.9%, respectively [13]. In another study in which two readers without extensive microbiology training used the same system to identify *S*. *aureus*, sensitivity ranged from 52% to 78% and specificity from 92% to 98% [12]. Likewise, other investigators used a different triplate for identification of Gram-negative bacteria, staphylococci, and other Gram-positive bacteria and achieved sensitivity and specificity for identification of *S*. *aureus* of 65% and 94% [32]. In the present study, the use of Accumast resulted in sensitivity and specificity of 100% and 99.8% for identification of *S*. *aureus* under field conditions when compared to standard laboratory culture. These results were confirmed by our *in vitro* studies, in which ATCC strains of *S*. *aureus*, *S*. *chromogenes*, and *S*. *epidermidis* were plated in all sections of the Accumast plate and the growth of *S*. *aureus* colonies were of pink coloration and markedly different from the other two species of staphylococci. The high predictive value of this system compared with other on-farm culture systems can be partially explained by the clear difference in color between *S*. *aureus* and other staphylococci in Accumast, as opposed to the need of identification of more subtle differences in colony characteristics and β-hemolysis in other methods [13]. In fact, hemolysin production by *S*. *aureus* has been shown to be variable [33]. It is important to acknowledge that this particular species comprised only 1.3% of the pathogen prevalence in the study sample and further research is needed to confirm the findings presented here. Additionally, when compared to 16S rRNA, Accumast presented PPV and a Cohen's kappa coefficient of 0.89, considered almost perfect agreement [20] for all bacterial groups evaluated. Unfortunately the use of this technique as the gold standard does not allow for calculation of NPV, Sensitivity and Specificity, since only positive results from the test being evaluated are available for comparison. However, as mentioned before, bacterial characteristics often used for species identification such as hemolysin production have been shown to be variable. This inconsistency is not an issue when targeted sequencing of the 16S rRNA gene is performed, once this gene has been proved to be highly conserved among different phenotypes of the same bacterial species.

The specificity and NPV of Accumast for identification of *Staphylococcus* sp. were above 95%; however, its sensitivity and PPV were much smaller compared to that of *S*. *aureus*, which can be a limitation of the on-farm culture system evaluated here. Similarly, previous reports also observed a reduced sensitivity for discrimination between *S*. *aureus* and other staphylococci [12, 13]. Although not ideal, the reduced capacity of on-farm culture system to identify other *Staphylococcus* sp. has a smaller impact on its applicability in dairy herds when compared to the capacity of correctly identifying *S*. *aureus*. Coagulase-negative staphylococci (**CNS**) are considered pathogens of minor importance compared with other bacteria while *S*. *aureus* remain a major concern because of its contagious behavior [34–36]. In fact, CNS has been associated with subclinical or moderate clinical mastitis and with high spontaneous cure rates [37–40]. Other studies argue that CNS are the main species responsible for mammary gland infection in ruminants, causing significant changes in milk metabolites that play an important role in the quality of dairy products [41, 42]. Regardless of the effect of CNS in mammary infections, milk yield, and downstream milk quality, we acknowledge that improvement on the capability of correctly diagnosing *Staphylococcus* sp. would be advantageous for the on-farm culture system presented here.

Environmental streptococci were the most prevalent bacteria isolated in the present study, which is in agreement with previous studies [7, 13, 26, 43]. Among environmental pathogens, *S*. *uberis* plays an important role in intramammary infections because of its invasive comportment and association with recurring infections [23, 29, 44]. The ability to identify cows infected with *Streptococcus* sp. is critical for health management in dairy herds as these infections respond well to commercially available intramammary antimicrobials [8]. The use of Accumast resulted in high overall sensitivity and specificity for identification of environmental streptococci independent of the species characterized by standard laboratory culture. The sensitivity and overall accuracy of Accumast plates for identification of *Streptococcus* sp. were comparable to the ones reported for the methods evaluated by McCarron et al. [43] and Royster et al. [12]. Although the differentiation among *Streptococcus* species using Accumast was not attempted, visual inspection of ATCC cultures indicate a lighter blue associated with *S*. *agalactiae* and *S*. *dysgalactiae* compared with that of *S*. *uberis*. Similar patterns were also observed during the field study; however, such nuances in tonality were not recorded and further research is necessary to evaluate the predictive values of Accumast for differentiation of *Streptococcus* sp. in the species level.

Bovine mastitis associated with *E*. *coli* has been reported to have high self-cure rates. In an elegant review, Suojala et al., (2013) compiled data from studies that evaluated the treatment of *E*. *coli* caused bovine mastitis and concluded that intramammary antibiotic therapy should not be recommended in mild and moderate cases [9]. For this reason, identifying mastitic cows infected primarily with *E*. *coli* is a critical step towards reducing the use of antibiotics in dairy herds. On the other hand, results reported by Schukken et al., (2011) support the use of intramammary antimicrobials for treatment of mild and moderate cases of Gram-negative mastitis. In that study, a randomized clinical trial was conducted and revealed a significant increase in the odds of clinical and bacteriological cure in treated animals when compared to non-treated controls [45]. The accuracy of Accumast to identify *E*. *coli* was 95.7% compared with standard laboratory culture, with sensitivity and specificity of 75.0% and 97.9%, respectively which are greater than the results from Viora et al. [32] that reported a sensitivity and specificity of 67% and 92% for identification of *E*. *coli* in the milk of mastitic cows using a triplate containing selective culture media. Nevertheless, the use of systemic antimicrobial therapy combined with support therapy and anti-inflammatory drugs are recommended in severe cases of *E*. *coli* mastitis due to the high indices of bacteremia experienced in cows undergoing this presentation of the disease [46, 47].

## Conclusions

The on-farm culture system evaluated in the present study is suitable for use under field conditions and presented substantial overall accuracy for detection of common mastitis pathogens, which was confirmed by 16S rRNA gene sequencing. Accumast provides a unique approach for on-farm identification of mastitis associated pathogens, mostly through its straightforward color-based classification of bacteria. Identification of bacteria based on color allows for easy interpretation by individuals with limited microbiological training; thus, providing the basis for selective antimicrobial therapy of mastitic cows based on causal microorganisms. Further research is warranted to evaluate test characteristics of Accumast between multiple study sites with distinct mastitis pathogens prevalence profiles and among readers without microbiology experience.

## Supporting Information

S1 File Detailed information about test results obtained from gold standard and on-farm culture in study 1. Table A represents True Positives, True Negatives, False Positives and False Negatives according to standard laboratory culture. Table B contains the distribution of results between on-farm culture system and standard laboratory culture.
(DOCX)
