# Supplementary material for: Evaluation of an On-Farm Culture System (Accumast) for Fast Identification of Milk Pathogens Associated with Clinical Mastitis in Dairy Cows
Source: PLoS One. 2016 May 13;11(5):e0155314. doi: 10.1371/journal.pone.0155314 (PMC4866694; doi:10.1371/journal.pone.0155314)
Supplement: S1 File — Table A represents True Positives, True Negatives, False Positives and False Negatives according to standard laboratory culture. Table B contains the distribution of results between on-farm culture system and standard laboratory culture. (DOCX) [file pone.0155314.s001.docx]

**S1 File Detailed information about test results obtained from gold standard and on-farm culture in study 1**

| **Table A.** Standard laboratory culture was considered the gold standard (study 1) | | | | |
| --- | --- | --- | --- | --- |
| **Accumast result** | **False**  **Negatives** | **False**  **Positives** | **True**  **Negatives** | **True**  **Positives** |
| *E. coli* | 4 | 2 | 0 | 23 |
| *E. coli, Staphylococcus* | 0 | 0 | 0 | 2 |
| *E. coli, Streptococcus* | 1 | 0 | 0 | 17 |
| *Enterococcus* | 1 | 0 | 0 | 0 |
| *Enterococcus, Staphylococcus* | 1 | 0 | 0 | 0 |
| *Negative* | 42 | 0 | 160 | 0 |
| Other Gram- | 1 | 1 | 0 | 9 |
| Other Gram-, *Staphylococcus* | 0 | 0 | 0 | 1 |
| Other Gram-, *Streptococcus* | 0 | 0 | 0 | 4 |
| *Staphylococcus* | 3 | 9 | 0 | 18 |
| *Pseudomonas* | 1 | 0 | 0 | 1 |
| *S. aureus* | 0 | 1 | 0 | 7 |
| *Streptococcus* | 8 | 5 | 1 | 194 |
| *Streptococcus, Staphylococcus* | 0 | 0 | 0 | 12 |
| Total | 62 | 18 | 161 | 288 |

^1^Only sections with more than five colonies were considered positive.

^2^ Contaminated samples in either standard culture (n = 3) or on-farm culture (n = 6) were not included in the analysis.

| **Table B.** Distribution of results between on-farm culture system and standard laboratory culture (study 1) | | | | | | | | | | | | | | | | | | | | | |
| --- | --- | --- | --- | --- | --- | --- | --- | --- | --- | --- | --- | --- | --- | --- | --- | --- | --- | --- | --- | --- | --- |
|  | **Standard laboratory culture performed by the Quality Milk Production Services laboratory** | | | | | | | | | | | | | | | | | | | | |
| **Accumast result**^1^ | *A pyogenes* | *E. coli* | *Enterococcus* | G- *bacillus* | *Klebsiella* | Negative | *Pseudomonas* | *S. aureus* | *Staph* sp | *S. dysgalactiae* | *S.dysgalactiae, S. uberis* | *Strep sp* | *Strep* sp*, E coli* | *Strep sp,*  *G- bacillus* | *Strep sp,*  *Klebsiella* | *Strep sp,*  *Staph sp* | *Strep sp, Strep dysgalactiae* | *Strep uberis* | *Strep uberis,*  *E coli* | *Strep uberis,*  *G- bacillus* | Total |
| *E. coli* | 0 | 23 | 0 | 2 | 2 | 2 | 0 | 0 | 0 | 0 | 0 | 0 | 0 | 0 | 0 | 0 | 0 | 0 | 0 | 0 | 29 |
| *E. coli*  *Staphylococcus* | 0 | 2 | 0 | 0 | 0 | 0 | 0 | 0 | 0 | 0 | 0 | 0 | 0 | 0 | 0 | 0 | 0 | 0 | 0 | 0 | 2 |
| *E. coli*  *Streptococcus* | 0 | 8 | 0 | 1 | 0 | 0 | 0 | 0 | 0 | 0 | 0 | 0 | 1 | 1 | 1 | 0 | 0 | 0 | 5 | 1 | 18 |
| *Enterococcus* | 0 | 0 | 0 | 0 | 0 | 0 | 0 | 0 | 0 | 0 | 0 | 0 | 0 | 0 | 0 | 0 | 0 | 1 | 0 | 0 | 1 |
| *Enterococcus*  *Staphylococcus* | 0 | 0 | 0 | 0 | 0 | 0 | 0 | 0 | 0 | 0 | 0 | 0 | 0 | 0 | 0 | 0 | 0 | 1 | 0 | 0 | 1 |
| Negative | 9 | 11 | 0 | 1 | 1 | 151 | 0 | 0 | 8 | 5 | 0 | 5 | 0 | 0 | 0 | 0 | 0 | 11 | 0 | 0 | 202 |
| Other Gram- | 0 | 1 | 0 | 0 | 9 | 1 | 0 | 0 | 0 | 0 | 0 | 0 | 0 | 0 | 0 | 0 | 0 | 0 | 0 | 0 | 11 |
| Other Gram- *Staphylococcus* | 0 | 0 | 0 | 0 | 0 | 0 | 0 | 0 | 1 | 0 | 0 | 0 | 0 | 0 | 0 | 0 | 0 | 0 | 0 | 0 | 1 |
| Other Gram-*Streptococcus* | 0 | 0 | 0 | 0 | 3 | 0 | 0 | 0 | 0 | 0 | 0 | 0 | 0 | 0 | 0 | 0 | 0 | 1 | 0 | 0 | 4 |
| *Staphylococcus* | 0 | 0 | 0 | 0 | 0 | 9 | 0 | 0 | 17 | 0 | 0 | 3 | 0 | 0 | 0 | 1 | 0 | 0 | 0 | 0 | 30 |
| *Pseudomonas* | 0 | 0 | 0 | 1 | 0 | 0 | 1 | 0 | 0 | 0 | 0 | 0 | 0 | 0 | 0 | 0 | 0 | 0 | 0 | 0 | 2 |
| *S. aureus* | 0 | 0 | 0 | 0 | 0 | 0 | 0 | 7 | 1 | 0 | 0 | 0 | 0 | 0 | 0 | 0 | 0 | 0 | 0 | 0 | 8 |
| *Streptococcus* | 1 | 1 | 7 | 0 | 0 | 5 | 0 | 0 | 0 | 31 | 1 | 45 | 0 | 0 | 0 | 0 | 1 | 116 | 0 | 0 | 208 |
| *Streptococcus*  *Staphylococcus* | 0 | 0 | 0 | 0 | 0 | 0 | 0 | 0 | 1 | 3 | 0 | 3 | 0 | 0 | 0 | 1 | 0 | 4 | 0 | 0 | 12 |
| All | 10 | 46 | 7 | 5 | 15 | 168 | 1 | 7 | 28 | 39 | 1 | 56 | 1 | 1 | 1 | 2 | 1 | 134 | 5 | 1 | 529 |

^1^Only sections with more than five colonies were considered positive.

^2^ Contaminated samples in either standard culture (n = 3) or on-farm culture (n = 6) were not included in the analysis.
